# Minor salivary gland tumours: Malignant or benign? A 10-year local retrospective review

**DOI:** 10.4102/jcmsa.v3i1.126

**Published:** 2025-02-17

**Authors:** Johan Grobbelaar, Kathryne E. Wright, Ann Thomas, Shaun E. Adam

**Affiliations:** 1 Department of Otorhinolaryngology, Faculty of Medicine and Health Sciences, Stellenbosch University, Cape Town, South Africa; 2 Department of Otorhinolaryngology, Faculty of Health Sciences, University of Cape Town, Cape Town, South Africa

**Keywords:** minor salivary gland tumours, salivary gland tumours, malignant, benign, pleomorphic adenoma, polymorphous adenocarcinoma

## Abstract

**Background:**

This study reports on our local incidence of minor salivary gland tumours (MSGT).

**Methods:**

This was a retrospective descriptive study including all patients with MSGT treated at Tygerberg Hospital from 2015 to 2024.

**Results:**

Benign and malignant tumours’ incidence was almost equal at 46.46% and 53.54%, respectively (*N* = 99). Pleomorphic adenomas were the most common tumour overall (38.38%) and the most common benign tumour. Polymorphous low-grade adenocarcinoma (PLGA) was the most common malignant tumour (15.15%). The palate was the most common site. Primary sites other than lips and palate had a 93.23% malignant incidence. Benign tumours occurred statistically significantly in younger patients than malignant tumours (*p* < 0.001) (42.74 years versus 56.02 years).

**Conclusion:**

We did not see the high malignant incidence that is frequently reported in otorhinolaryngology textbooks. Pleomorphic adenomas remain the most common and occur most commonly in the palate. We did show an unusually high incidence of PLGA. Primary sites other than palate and lips should raise the suspicion of malignant MSGT.

**Contribution:**

The study highlights the importance of reporting on local data because disease patterns may vary greatly based on genetic and geographical differences. Further investigation is needed to explain possible local factors contributing to the high incidence of PLGA.

## Introduction

This study primarily aimed to report on the benign versus malignant incidence and secondarily on the different histopathology of minor salivary gland tumours (MSGT). Major salivary glands in the head and neck are the parotid, submandibular and sublingual glands, found bilaterally. Minor salivary glands are located throughout the upper aero digestive tract but are most commonly found on the hard palate, followed by the buccal mucosa, retro-molar trigone, and lips. Salivary gland tumours only account for 3% – 10% of all head and neck tumours.^1^ Minor salivary gland tumours are uncommon and only comprise 9% – 23% of salivary gland tumours.^2^

Classical teaching stipulates that the chance of a salivary gland tumour being malignant increases from major to minor salivary glands.^3^ Dictums such as 80% of salivary gland tumours occur in the parotid glands, 80% of parotid gland tumours are benign, and 80% of benign parotid gland tumours are pleomorphic adenomas (PA) are commonly taught.^4^ The inverse is taught with regard to MSGT, with malignancy rates varying from 50% to 80%.^4^ Two leading textbooks report the malignant incidence as indicated in Table 1.

**TABLE 1:** Incidence of salivary gland tumours being malignant.

Gland	Scott-Brown^4,13^ malignant (%)	Cummings^1^ malignant (%)
Parotid	15 – 32	25
Submandibular	41 – 45	43
Sublingual	70 – 90	-
Minor salivary glands	80	85

Note: Please see the full reference list of the article, Grobbelaar J, Wright KE, Thomas A, Adam SE. Minor salivary gland tumours: Malignant or benign? A 10-year local retrospective review. J Coll Med S Afr. 2025;3(1), a126. https://doi.org/10.4102/jcmsa.v3i1.126, for more information.

Articles differ in their reported incidence of benign versus malignant tumours. A frequently quoted study from M.D. Anderson cancer centre reported a malignant incidence of 78%, as well as a recent study from India with a reported 75% malignant incidence.^5,6^ However, in direct contrast to this, a recently published article in a Chinese population reported a malignant incidence of only 45%.^7^

Only two previous studies reported on local patterns in South Africa. Firstly, Isacsson et al. reported on 201 patients from 1958 to 1979 who presented to the University of the Witwatersrand. In their series, the malignant incidence was 28%.^8^ Secondly, van Heerden et al. reported on 70 patients from Sefako Makgatho Health Sciences University (previously Medunsa). They found a malignant incidence of 52%.^9^ It is clear from the literature review that there are huge geographical differences in the rates of malignant incidence, and some studies suggest that there might even be ethnic differences.^6^

## Research methods and design

This was a retrospective descriptive study. All patients with MSGT treated at Tygerberg Hospital, Cape Town, South Africa from 2015 to 2024 were included. Tygerberg Hospital is one of the two major referral hospitals dealing with head and neck cancers in the Western Cape and is the training hospital affiliated with the Faculty of Medicine and Health Sciences, Stellenbosch University, South Africa.

Cases were identified by conducting a manual search of the surgery register book to identify patients who presented with salivary gland tumours, and then more specifically, those with MSGT. We also used the databases of the Department of Oral and Maxillofacial Pathology, University of the Western Cape and the National Health Laboratory Services (NHLS).

A manual process was followed to include patients who had fine needle aspiration, punch, core needle, or excisional confirmed MSGT. Duplicate files were excluded, as well as any histology performed at other laboratory services.

IBM^®^ Statistical Package for Social Sciences (SPSS) version 29.0 was used to analyse the data. Frequency tables and percentages were reported to describe nominal or binary data. Chi-squared tests were used to compare proportions between groups. Continuous data were summarised using mean and standard deviation and compared between groups using *t*-tests.

### Ethical considerations

Ethical approval was obtained from the Health Research Ethics Committee (HREC) from Stellenbosch University (N23/08/108) and Tygerberg Hospital National Health Research Database (WC_202310_015).

## Results

We report on 99 patients. The malignant incidence was 53.54% (95% CI: 43.27% – 63.52%). Overall, the most common tumour was pleomorphic adenoma (38.38%), followed by polymorphous low-grade adenocarcinoma (PLGA) (15.15%), and adenoid cystic carcinoma (ACC) (14.14%). Table 2 gives a breakdown of all the MSGT according to frequency seen.

**TABLE 2:** Histological types, incidence, and their percentage of the total benign or malignant group.

Histological type	Incidence (%)	Malignant or benign	Malignant or benign group (%)
Pleomorphic adenoma (PA)	38.38	Benign	82.61
Polymorphous low-grade adenocarcinoma (PLGA)	15.15	Malignant	28.30
Adenoid cystic carcinoma (ACC)	14.14	Malignant	26.42
Adenocarcinoma (AdC)	8.08	Malignant	15.09
Muco-epidermoid carcinoma (MEC)	6.06	Malignant	11.32
Myoepithelioma (ME)	4.04	Benign	8.70
Acinic cell carcinoma	3.03	Malignant	5.66
Secretory carcinoma	2.02	Malignant	3.77
Myoepithelial carcinoma	2.02	Malignant	3.77
Epithelial myo-epithelial carcinoma	1.01	Malignant	1.89
Lymphoepithelial-like carcinoma	1.01	Malignant	1.89
Salivary duct carcinoma	1.01	Malignant	1.89
Papillary cystadenoma	1.01	Benign	2.17
Secretory adenoma	1.01	Benign	2.17
Basal cell adenoma	1.01	Benign	2.17
Canalicular adenoma	1.01	Benign	2.17

The most common sites affected were the hard palate (33.33%), soft palate (19.19%), and palate not otherwise specified (NOS) (13.13%). Table 3 gives a breakdown of all the anatomical sites affected and benign versus malignant incidence of any MSGT in that site.

**TABLE 3:** Anatomical sites and benign versus malignant incidence.

Site	Incidence (%)	Benign (%)	Malignant (%)
Hard palate	33.33	45.45	54.55
Soft palate	19.19	68.42	31.58
Palate NOS	13.13	61.54	38.46
Buccal mucosa	12.12	41.67	58.33
Maxillary sinus	8.08	12.50	87.50
Lip	4.04	100.00	-
Oral tongue	3.03	-	100.00
FOM	2.02	-	100.00
RMT	2.02	-	100.00
BOT	1.01	-	100.00
Nasopharynx	1.01	-	100.00
Tonsil	1.01	-	100.00

NOS, not otherwise specified; FOM, floor of mouth; RMT, retro-molar trigone; BOT, base of tongue.

Overall, the average age was 50.19 years (10–84 years). The average age for benign tumours was significantly lower at 42.74 years (standard deviation [s.d.]: ±19.9) compared to malignant tumours of 56.02 years (s.d.: ±15.1) (*p* < 0.001). This is also in keeping with published data, as shown in Table 4, confirming that benign tumours occur earlier than malignant tumours.^5,6,7,9^

**TABLE 4:** Average age of minor salivary gland tumours – Benign versus malignant.

Tumour pathology	Average age in years (s.d.)				
	Van Heerden^9^	Beckhardt^5^	Wang^7^	Vani^6^	Current study
Benign MSGT	36.5 ± 14.7	51	47.58 ± 16	40.36 ± 13.44	42.74 ± 19.9
Malignant MSGT	49.8 ± 16.3	56	51.51 ± 14.19	48.06 ± 13.79	56.02 ± 15.1

Note: Please see the full reference list of the article, Grobbelaar J, Wright KE, Thomas A, Adam SE. Minor salivary gland tumours: Malignant or benign? A 10-year local retrospective review. J Coll Med S Afr. 2025;3(1), a126. https://doi.org/10.4102/jcmsa.v3i1.126, for more information.

MSGT, minor salivary gland tumours; s.d., standard deviation.

In our series, females accounted for 56.57% of all MSGTs, giving a male: female ratio of 1:1.3, which is very much in keeping with published data.^6,7^ Although 56.57% of all the tumours occurred in females, there was no sex difference with regard to benign or malignant pathology (*p* = 0.678).

## Discussion

Minor salivary gland tumours are rare and reporting on them has inherent problems. Some of the main problems are reclassifications of tumours, inter observer variability, geographic variation, and referral bias.^10^ However, almost none of the referral hospitals in our drainage area will offer surgery to these patients; therefore, we feel that our data are a true reflection of the incidence rates.

We did see important findings, some supporting published data and others showing important loco-regional variation from international data. To answer our primary question, our local data showed an almost equal incidence of benign versus malignant MSGT. Therefore, taking the only other two studies from South Africa into account,^8,9^ there is not a high incidence of malignancy rate that is frequently quoted internationally.

The results showed that 65.66% of all MSGT occurred in the palate. Of all the MSGT occurring in the palate, 56.94% were benign and 43.06% malignant tumours, respectively. The palate was the primary site of 78.26% of all benign tumours, but only 54.72% of malignant tumours. This is in contrast to a clinical review of MSGT which reported that 55% of all MSGT occurred in the palate and of those, 60% were malignant.^10^ Interestingly, our results showed that once the palate and lips are excluded, the chance of it being a malignant tumour increases to 92.23%. In fact, MSGT occurring in the oral tongue, floor of mouth, retro-molar trigone, nasopharynx, and tonsil were all malignant; however, the numbers were low. Furthermore, 87.50% of maxillary sinus MSGT were malignant.

Our six most common tumours, which comprised 85.85% of all tumours, are summarised in Table 5. All tumours occurred most commonly in the palate, except for ACC. Adenoid cystic carcinomas occurred almost equally in the palate (35.71%) and maxillary sinus (28.57%). The average age of patients with PA was 40.68 years (10–83 years). This was significantly lower compared to PLGA (59.53 years), ACC (57.31 years), and adenocarcinomas (AdC) (57.25 years). Interestingly, the average age of patients with muco-epidermoid carcinomas (MEC) was 43.33 years and all of them occurred in females. As shown in Table 5, there was a female preponderance in PA, PLGA, and MEC. In contrast, ACC, AdC, and myoepitheliomas (ME) were more common in males.

**TABLE 5:** Comparative data of this study.

Epidemiology	PA	PLGA	ACC	AdC	MEC	ME
Average age (years)	40.68	59.53	57.31	57.25	43.33	49
Average age range (years)	10–83	36–81	26–72	27–72	28–71	24–62
M:F ratio	1:1.1	1:4	1.6:1	3:1	All females	3:1
Palate as primary site (%)	82.05	66.67	35.71	62.5	50	75

PA, Pleomorphic adenoma; PLGA, polymorphous low-grade adenocarcinomas; ACC, adenoid cystic carcinomas; AdC, adenocarcinomas; MEC, muco-epidermoid carcinomas; ME, myoepitheliomas; M, male; F, female.

Polymorphous low-grade adenocarcinoma, now just referred to as polymorphous adenocarcinoma, was the most common malignant tumour in our series. It comprised 15.15% of all tumours and 28.30% of the malignant group. This is extremely atypical when compared to international data. Van der Poorten et al. in their clinical review including 16 studies, stated that ACC accounts for 32% – 71% and MEC 15% – 38% of all MSGT and combined are by far the most common histological subtype found in MSGT (79%).^10^ They, nor textbooks, give an incidence of PLGA and it is accepted to be in the lower single digits.^10,11,12^ Two studies that do report on PLGA give an overall incidence of 2.47% and 9.73%, respectively, and a percentage of all malignant MSGT of 5.53% and 13.04%, respectively.^6,7^ However, van Heerden et al. from South Africa found an almost identical incidence of 15.7% of all MSGT and 30% of the malignant group.^9^ In their study, it was also the most common malignant tumour, and this possibly points to a geographical trend in South Africa. Another interesting finding is the low incidence of MEC, namely 6.06% of all MSGTs, and all of them occurred in females. This has not been reported elsewhere.

## Conclusion

Minor salivary gland tumours remain rare. In our setting, the incidence of malignant versus benign tumours is almost equal. Tumours occur most commonly in the palate and are mostly PA. The incidence of muco-epidermoid and ACC is low in our series and that of polymorph low-grade adenocarcinoma unusually high. Primary sites other than palate and lips should raise the suspicion of a malignant MSGT.
